# AZU1 (HBP/CAP37) and PRKCG (PKC-gamma) may be candidate genes affecting the severity of acute mountain sickness

**DOI:** 10.1186/s12920-023-01457-3

**Published:** 2023-02-20

**Authors:** Zhichao Xu, Qiong Li, Xiaobing Shen

**Affiliations:** 1 Key Laboratory of Environmental Medicine Engineering, Ministry of Education, School of Public Health, Southeast University, Nanjing, Jiangsu Province China; 2 Department of Epidemiology and Health Statistics, School of Public Health, Southeast University, Nanjing, Jiangsu Province China

**Keywords:** Acute Mountain sickness, High-altitude, WGCNA, Candidate genes, Azurocidin 1, Protein kinase C gamma

## Abstract

**Background:**

Acute Mountain Sickness (AMS) is one of the diseases that predispose to sudden ascent to high altitudes above 2500 m. Among the many studies on the occurrence and development of AMS, there are few studies on the severity of AMS. Some unidentified phenotypes or genes that determine the severity of AMS may be vital to elucidating the mechanisms of AMS. This study aims to explore the underlying genes or phenotypes associated with AMS severity and to provide evidence for a better understanding of the mechanisms of AMS.

**Methods:**

GSE103927 dataset was downloaded from the Gene Expression Omnibus database, and a total of 19 subjects were enrolled in the study. Subjects were divided into a moderate to severe AMS (MS-AMS, 9 subjects) group and a no or mild AMS (NM-AMS, 10 subjects) group based on the Lake Louise score (LLS). Various bioinformatics analyses were used to compare the differences between the two groups. Another dataset, Real-time quantitative PCR (RT-qPCR), and another grouping method were used to validate the analysis results.

**Result:**

No statistically significant differences in phenotypic and clinical data existed between the MS-AMS and NM-AMS groups. Eight differential expression genes are associated with LLS, and their biological functions are related regulating of the apoptotic process and programmed cell death. The ROC curves showed that AZU1 and PRKCG had a better predictive performance for MS-AMS. AZU1 and PRKCG were significantly associated with the severity of AMS. The expression of AZU1 and PRKCG were significantly higher in the MS-AMS group compared to the NM-AMS group. The hypoxic environment promotes the expression of AZU1 and PRKCG. The results of these analyses were validated by an alternative grouping method and RT-qPCR results. AZU1 and PRKCG were enriched in the Neutrophil extracellular trap formation pathway, suggesting the importance of this pathway in influencing the severity of AMS.

**Conclusion:**

AZU1 and PRKCG may be key genes influencing the severity of acute mountain sickness, and can be used as good diagnostic or predictive indicators of the severity of AMS. Our study provides a new perspective to explore the molecular mechanism of AMS.

**Supplementary Information:**

The online version contains supplementary material available at 10.1186/s12920-023-01457-3.

## Background

Acute Mountain Sickness (AMS) has defined as a syndrome involving headache, dizziness, gastrointestinal symptoms, insomnia and fatigue after arrival at a high altitude (> 2500 m) [1]. The disease occurs in people who have lived on the plains for a long time and have been exposed to high-altitude areas for a short period. Many factors contribute to AMS, and acute hypobaric hypoxia may serve as a principal etiological factor for Acute Mountain Sickness (AMS) [2]. In China, about 15 million people go to high-altitude for work or travel every year, and the incidence rate of AMS is almost 43%~69% [3, 4]. Besides, AMS may progress to high altitude pulmonary edema, high altitude cerebral edema, and even death [5, 6]. Therefore, AMS has become a public health problem of increasing concern [7].

Multiple factors influence the occurrence of AMS. In addition to environmental factors, for the population susceptibility, AMS occurs mostly in the male population [8]. Being overweight or obese is another factor contributing to the occurrence of AMS [9]. In terms of individuals, many clinical phenotypes were changed at high altitudes. The partial pressure of arterial oxygen decreases for a short period, resulting in lower arterial oxygen saturation [10]. Slowly, the bioavailability of NO decreases, leading to pulmonary vasoconstriction and consequent high-altitude pulmonary edema [11]. Under prolonged hypoxic conditions, hemoglobin production increases, increasing blood viscosity and leading to altitude erythrocytosis [12]. While some changes benefit athletes in terms of improved athletic performance, they are detrimental to most of the general population exposed to high altitudes for short periods [13].

Previous studies have shown that the hypoxia-inducible factor (HIF) pathway plays a significant role in hypoxic adaptation [14]. Under hypoxic conditions, hydroxylation and degradation of HIF are inhibited, and HIF induces transcription of downstream genes by binding to hypoxia response elements (HREs) [15]. For example, HIF regulates vascular endothelial growth factor (VEGF) [16]. VEGF promotes endothelial cells to add value and form new blood vessels, which facilitates the improvement of the ventilation-perfusion ratio (V/Q) to adapt to the hypoxic environment [17, 18]. Erythropoietin (EPO) is regulated by HIF and promotes erythropoiesis [19]. Many related genes, such as PPAR, NF-kb, p53, and P13K, are part of pathways that function together to adapt to the hypoxic environment [20].

However, few studies have focused on phenotypes or genes associated with AMS severity, especially in the early stages of AMS. The discovery of more phenotypes or genes associated with the severity of AMS may lead to a better understanding of the mechanisms of AMS occurrence and development, a variable that distinguishes the severity of AMS can guide the use of clinical medications and reduce the incidence of death due to AMS. Therefore, phenotypic or gene expression differences between no or mild AMS and moderate to moderate AMS deserve to be explored.

Since 1993, the diagnosis and severity evaluation of AMS relies on the Lake Louise Score (LLS) [21]. LLS is a subjective judgment by the study participants of their symptoms of headache, gastrointestinal symptoms of fatigue, and dizziness [22]. People rate the severity of each symptom (0–3) based on how they feel, and the sum of the symptom scores is the total LLS score. Although there have been many debates on LLS in recent years, such as the mental state of the subject at sea level and the specificity of LLS, LLS remains one of the criteria for rapid diagnosis of AMS until a conclusion is reached [23–25]. The AMS-C is one of the indicators included in the Environmental Symptoms Questionnaire (ESQ) and, in recent studies, is another criterion widely used to diagnose AMS [26–28]. In this study, we use AMS-C grouping to validate the analysis results derived from LLS grouping to make the analysis results more convincing.

Nowadays, more publicly available datasets are submitted to Gene Expression Omnibus (GEO) database with the development of technologies [29]. Transcriptome and microarray analysis have been used in various diseases, including tumors, AMS, and high altitude pulmonary edema (HAPE). Through bioinformatics analysis methods, such as weighted gene co-expression network analysis (WGCNA), the genes or phenotypes most associated with AMS can be identified to discover potential biomarkers and pathways associated with AMS, which are critical for disease diagnosis, treatment, and prevention.

Therefore, this study aimed to explore essential candidate genes and phenotypes for moderate-to-severe AMS to provide evidence for a better understanding of the mechanisms of AMS. The dataset GSE103927 and its original study in the GEO database contains general characteristics of the study subjects, clinical data, LLS, and transcriptome information. This dataset meets the needs of this study. We analyzed this dataset by bioinformatics methods such as differentially expressed gene screening and WGCNA. AZU1 and PRKCG were finally identified that were associated with AMS severity. This result was validated by an alternative grouping approach and clinical samples.

## Methods

### Data collection and study design

The datasets analyzed during the current study are available in the GEO repository, https://www.ncbi.nlm.nih.gov/geo/query/acc.cgi?acc=GSE103927. Dataset GSE103927 was downloaded from GEO database (https://www.ncbi.nlm.nih.gov/gds) by R software (version 4.0.4) on March 25, 2022. One hundred twelve total samples representing 21 subjects at 7-time points (not all subjects represented at all time points) were taken. Nineteen samples exposed to the high altitude at noon on the first day (H1) were included in this study. In this study, we focused on analyzing the data during the first day of exposure to high altitude (H1) and sea level (SL). However, in this dataset, at time point H1, transcriptome information was included for only 19 subjects. Therefore, the other 2 subjects were excluded from this study, and the total number of subjects in this study was 19.

**Fig. 1:**
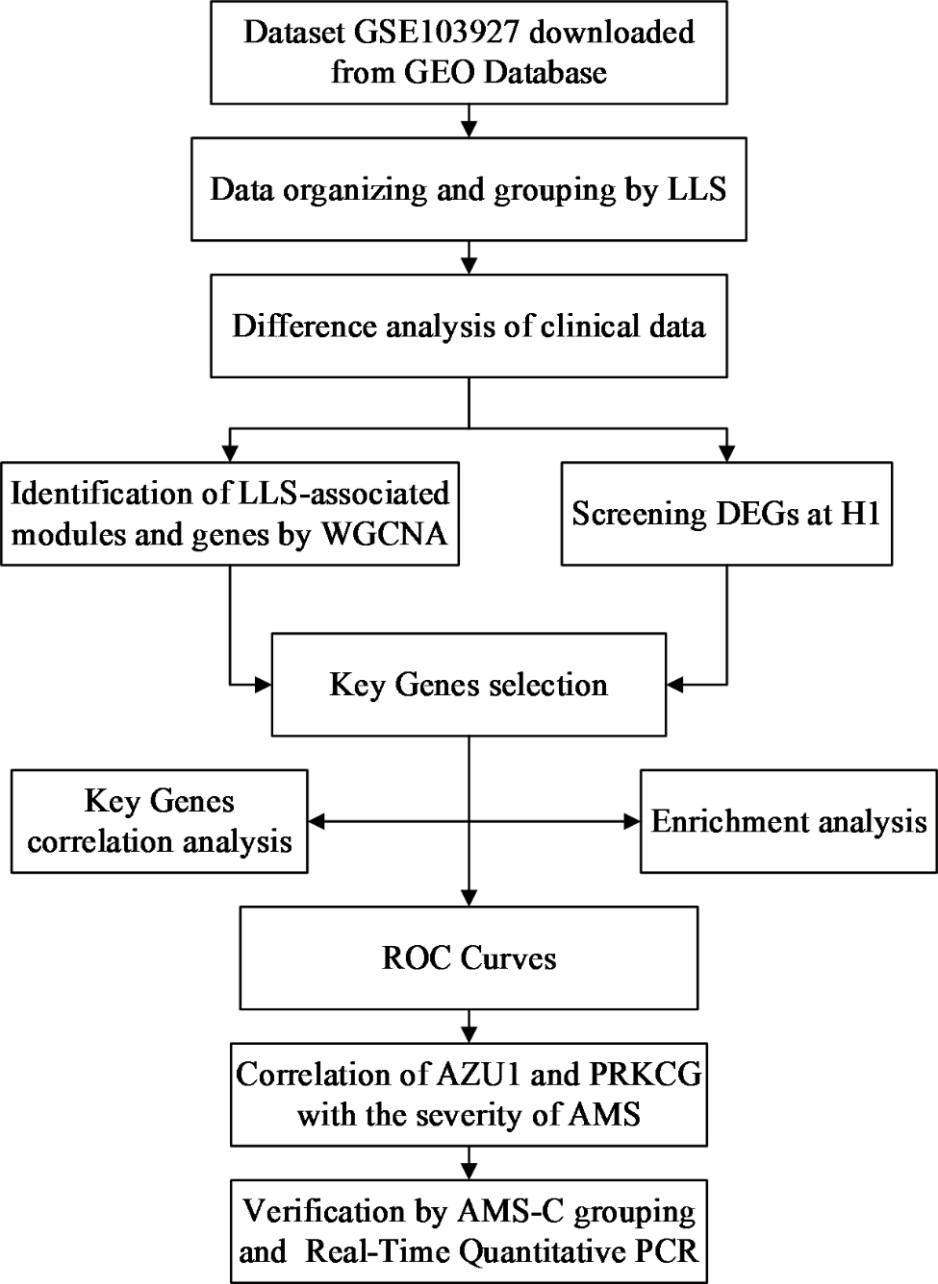
Flow chart of the study design

General characteristics and clinical data of subjects were obtained from the supplementary material of *Andrew*’s study [30]. Clinical data at other time points were excluded except for those measured at H1 and Sea Level (SL). Multiple imputation, an approach used to generate synthetic data that accurately represents group-level results, was used to impute missing values [31]. This process was implemented through the “mice” package in R software (version 4.0.4) [31–33]. Multiple interpolation was performed five times, and the final average was taken [34, 35]. The code is available in Table S1. Since all data used for analysis in this study were obtained from a publicly available database, Ethics Committee approval was not required. Figure 1 briefly illustrates our study design.

### Definition of acute mountain sickness and grouping

Based on the 2018 Lake Louise Score (LLS) criteria, AMS cases must have headache symptoms (headache score > 0), then a total score of 3–5 was defined as mild AMS, moderate AMS as 6–9 points, and severe AMS as 10–12 points [21]. Based on the latest LLS and previous study, subjects were divided into two groups, namely moderate to severe AMS (MS-AMS, 9 subjects’ LLS ≥ 6) and mild or no AMS (NM-AMS, 10 subjects’ LLS < 6) [36, 37]. According to AMS-C, subjects were classified into four categories, namely normal (AMS-C < 0.700), mild (0.700 ≤ AMS-C < 1.530), moderate (1.530 ≤ AMS-C < 2.630) and severe (AMS-C ≥ 2.630) [37]. All the details about the grouping can be seen in part 3.1. The NM-AMS-C group (9 subjects) included all subjects with an AMS-C < 1.530, and other subjects were included in the MS-AMS-C group (10 subjects). The grouping according to AMS-C will be applied in Sect. 3.7. Details of the grouping are shown in Table 1.

**Table 1:** The number of subjects in each group under different grouping methods

Grouping method	Normal	Mild	Moderate	Severe	NM-AMS (NM-AMS-C)	MS-AMS (MS-AMS-C)
LLS	3	7	7	2	10	9
AMS-C	3	6	6	4	9	10

### Difference analysis of clinical data and differentially expressed genes (DEGs)

The ‘limma’ package in R language software (version 4.0.4) based on a generalized linear model was used to analyze the DEGs [38]. DEGs with |Fold change|>1 and *P* value < 0.050 were screened in this research [39]. Use SangerBox (http://vip.sangerbox.com/) for graphing and visualizing the results using volcano and heatmap [40].

### Weighted gene co-expression network analysis (WGCNA)

The Median Absolute Deviation (MAD) of each gene was calculated, genes with MAD less than the median were excluded, outlier genes and samples were removed by the ‘goodSamplesGenes’ method of the R package ‘WGCNA’, and finally, a co-expression network was created for the genes in the MS-AMS group [41]. LLS was selected as a representative to identify modules and associated genes. Genes in modules associated with LLS are considered to be co-expressed LLS-related genes.

First, the Pearson correlation matrix and average linkage methods were performed for all pairs of genes. Then a weighted adjacency matrix is constructed using the power function A_mn=|C_mn|^β. β is a soft threshold parameter that emphasizes the strong linkage between genes and penalizes weak linkage. A suitable soft threshold of 18 was selected (Figure S1). The adjacency relationships are translated into a topological overlap matrix (TOM), which measures the network connectivity of a gene, and the corresponding dissimilarity (1-TOM) is calculated. To classify genes with similar expression profiles into gene modules, the average linkage hierarchical clustering was performed based on the TOM dissimilarity measure with a minimum gene dendrogram size (genome) of 30. The sensitivity is set to 3. To further analyze the modules, dissimilarities of module feature genes were calculated, a cut line was selected for the module dendrogram, and some modules were merged. Gene significance (GS) for LLS and modules membership (MM) were calculated. Five hundred eighty-seven genes with GS > 0.500 and MM > 0.800 of the modules significantly correlated with LLS were selected as key genes [40, 42].

### Candidate genes selection, correlation analysis, and differential expression analysis

Candidate genes were selected from the intersection of the DEGs in part 3.2 and the genes in part 3.3. The Wayne diagram visualizes the intersection. The ‘corrplot’ package in R software was used to explore correlations between candidate genes. The boxplot made by SangerBox (http://vip.sangerbox.com/login.html) was used to visualize the differences in the expression of candidate genes among different groups [40].

### Functional annotation and pathway enrichment analysis

For gene set functional enrichment analysis, the ‘org.Hs.eg.db’ package and the ‘clusterProfiler’ package in R language software were used for GO annotation of genes [43]. A minimum gene set of 30 and a maximum gene set of 5000 were set, and the P value of < 0.050 were considered statistically significant. KEGG (Kyoto encyclopedia of genes and genomes) pathway enrichment for key genes using online tools (https://david.ncifcrf.gov/) [44–46]. Visualize the results with a bar-plot, bubble-plot, and circle map [40].

### Receiver operating characteristic (ROC) Curves analysis and correlation analysis

ROC curves were used to evaluate the predictive ability of candidate genes on the severity of AMS. The ROC curve analysis was completed by the ‘ROCR’ package and ‘rms’ package in R language software. Correlation analysis was used to explore the relationship between candidate genes and the severity of AMS.

### Cell culture and hypoxia treatment

BEAS-2B cells were obtained from laboratory passaged cultures. Cells were maintained in DMEM basic (Gibco, USA) with a mixture of 10% fetal bovine serum (BI, China) and 1% penicillin-streptomycin (Gibco, USA). All cells were cultured and passaged at 37 °C, 5% CO_2_ before treatment was applied. Control cells were cultured at 37 °C and 5% CO_2_ for 24 h. Cells in the experimental group were treated with hypobaric hypoxia and cultured at 37 °C in 1 ± 0.3% O_2_, 5% CO_2_, and 94% N_2_ for 24 h.

### Real-time quantitative PCR

RNA was isolated using the RNA-easy isolation Reagent (Vazyme, China). Then reversed transcribe the RNA to cDNA according to the reverse transcription kit instructions (GeneStar, China). Real-time quantitative PCR (qRT-PCR) was completed using SYBR Green (GeneStar, China). The primers were synthesized by Sangon Biotech (Shanghai, China) and are shown in Table 2. PCR amplification was performed for 40 cycles using the following conditions: denaturation 95 °C for 15s, annealing 60 °C for 30s and extension 72 °C for 30s. The qRT-PCR results were evaluated using the ∆∆Ct method. Bar graphs present the results.

**Table 2:** Primers Sequence List

Genes	Forward primer 5’-3’	Reverse primer 5’-3’
AZU1	TGAGCGAGAATGGCTACGAC	GAGGCAGTGGCAGTATCGTC
PRKCG	AGCCACAAGTTCACCGCTC	GGACACTCGAAGGTCACAAAT
GAPDH	GCAGGGGGGAGCCAA AAGGG	TGCCAGCCCCAGCGTCAAAG

### Statistical analysis

SPSS software (version 26.0) was used to compare general characteristics and clinical data between different groups. LLS, AMS-C and differences between groups for each gene were analyzed by Mann-Whitney U. One-way ANOVA analysis was used for overall comparison between groups. Independent-Samples T-test was used to test statistically significant differences of other variables in the two groups. The filtering of DEGs is done through the ‘limma’ package in R language software (version 4.0.4). ROC curve analysis was performed using SPSS 26.0 and plotted using the ‘ROCR’ package and the ‘rms’ package in R. The ‘corrplot’ package in R was used to complete Pearson correlation analysis between key genes. Two-sided *P* value < 0.050 means the difference is statistically significant.

## Results

### Difference of general characteristics and clinical data

General characteristics and clinical data were obtained from previous research and its supplementary material. Missing values in these data were filled by multiple imputation [30, 33]. In general, 19 subjects (8 females) were included in this study.

We try to identify phenotypic or clinical differences related to the severity of AMS early in the disease, as this is what people tend to focus on. By comparing the general characteristics at H1, no statistically significant difference in gender was found between the NM-AMS group and the MS-AMS group (*Χ*^2^ = 0.398, *P* = 0.528). Besides, the differences between the two groups in other general characteristics were also not statistically significant (Table S2).

By comparing the clinical data at H1, the difference in HB between the NM-AMS group and MS-AMS group was close to statistical significance (t = 2.103, *P* = 0.052), and there was no statistically significant difference in general characteristics between the NS-AMS and MS-AMS groups (Table S3).

The results of the above analysis suggest to us that after subjects are exposed to a plateau environment and occur AMS, patients with moderate to severe AMS may not be identified in time solely by general characteristics or clinical data. Therefore, in the following section, we analyzed the gene expression of our subjects in order to explore variables that could detect or predict the severity of AMS in a timely manner.

### The LLS and DEGs between the two groups

**Fig. 2:**
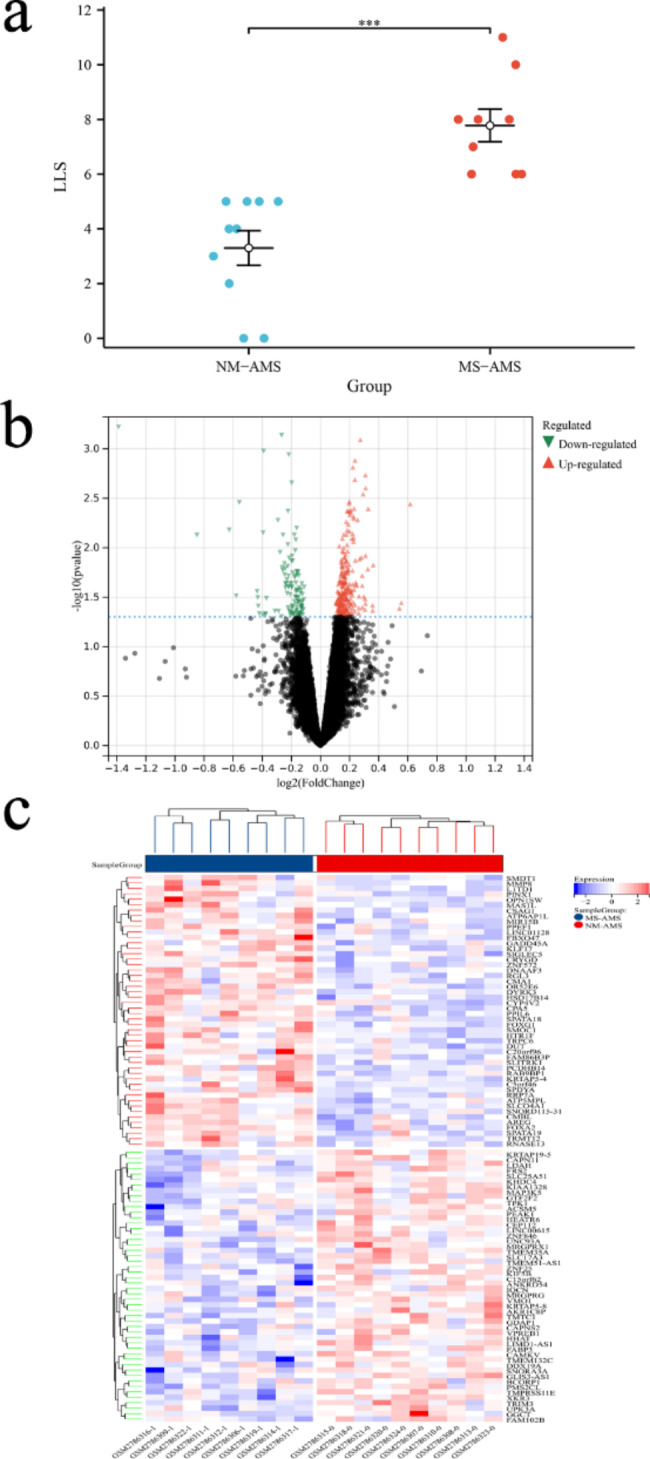
The LLS and DEGs between the two groups. (a) Differences in LLS between the MS-AMS group (9 subjects) and the NM-AMS group (10 subjects). (b)(c) The volcano plot and heatmap show DEGs in the MS-AMS group vs. the NM-AMS group. In total, 368 DEGs between the NM-AMS group and the MS-AMS group were identified, of which 252 were up-regulated, and 116 were down-regulated. LLS, lake louis score; DEGs, differentially expressed genes

There was a clear significant difference in LLS between the two groups, as shown in Fig. 2a. A total of 368 DEGs (252 were up-regulated and 116 were down-regulated) were identified between the NM-AMS group and the MS-AMS group, with the screening criteria of |Fold change|>1 and *P* < 0.050, the visualization result is shown in Fig. 2b, c [39]. This suggests to us that it may be the differential expression of these genes between the two groups that causes the difference in severity in AMS patients. However, the relationship between the expression of these DEGs and LLS is unknown. We will explore the genes associated with LLS in the next section.

### Discovering the most significant LLS-Related modules and genes

**Fig. 3:**
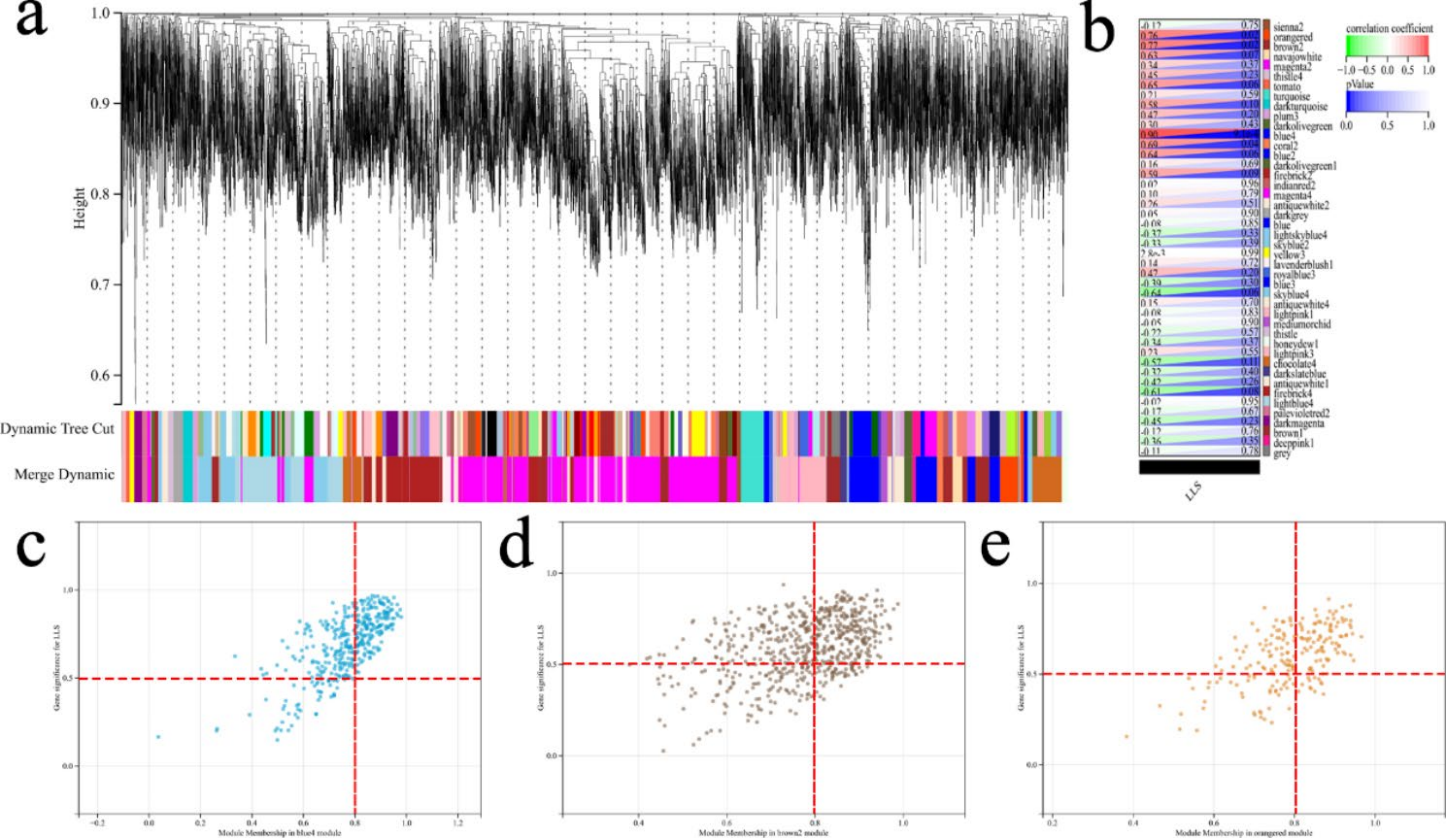
The Most Significant LLS-Related Modules and Genes. (a) Co-expression module identification in MS-AMS group. The branches of the cluster dendrogram represent the 44 different gene modules. Each module denoted a collection of co-related genes and was given a unique color. Each piece of the leaves on the cluster dendrogram represents a gene. (b) A heatmap shows the correlations and significant differences between the gene modules and LLS. The upper left corner of each cell displays R^2^, between 0 and 1. The *P*-value is displayed in the lower right corner of each cell. Significantly associated modules are blue4 (c), brown2 (d), and orangered (e), and the scatter plot of module characteristic genes is shown in Figure. Each circle represents a gene, and the genes in the upper right corner represent key genes for that module. LLS, lake louis score

After screening the DEGs, in order to find out the genes most related to LLS, a gene co-expression network was constructed through WGCNA. Each module contained a minimum of 30 genes, and the sensitivity was set to 3. Modules with a distance less than 0.25 were merged together, resulting in 44 modules, of which module blue4 (*R*^2^ = 0.900, *P* < 0.001), module brown2 (*R*^2^ = 0.770, *P* = 0.020), and module orangered (*R*^2^ = 0.760, *P* = 0.020) were the most relevant to LLS (Fig. 3a, b). A heat map showing the clustering of module feature vectors was made and can be seen in the attachment (Figure S2). All three modules were positively correlated with LLS.

The selection criteria for key genes were: the module in which the gene was located was significantly correlated with LLS, GS > 0.500 and MM > 0.800, and weighting threshold ≥ 0.100. As shown in Fig. 3c, d, **and e**. According to this principle, 530 genes in the three modules were considered key genes when LLS was considered.

### Candidate genes selection, correlation analysis, and differential expression analysis

**Fig. 4:**
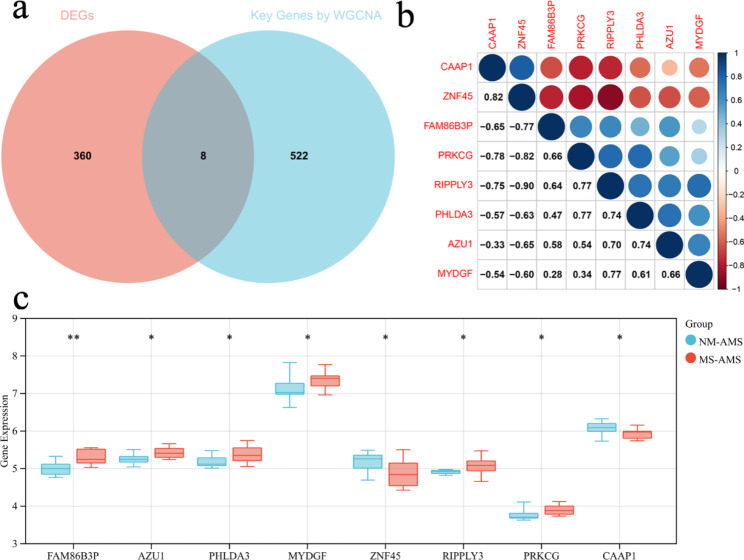
Candidate Genes Selection, Correlation analysis, and Differential Expression analysis. (a) A Venn diagram showing the number of DEGs versus the number of Key Genes obtained by WGCNA repeats. (b) Differential expression of candidate genes among different groups. (c) Candidate gene correlation. The lower left part shows the correlation coefficient, and the size of the circle in the upper right part is consistent with the size of the absolute value of the correlation coefficient; the more significant the absolute value of the correlation coefficient, the larger the graph is, and the darker the color. Red represents negative correlation, and blue represents positive correlation, as shown in the legend on the right. DEGs, differentially expressed genes; WGCNA, weighted gene co-expression network analysis. *, *P* < 0.050; **, *P* < 0.010

Genes in both DEGs and Key Genes obtained by WGCNA were selected as candidate genes. Figure 4a visualizes this result. The final 8 genes selected were CAAP1, ZNF45, FAM86B3P, PRKCG, RIPPLY3, PHLDA3, AZU1, and MYDGF. These eight genes are both associated with LLS and differentially expressed genes, suggesting that they are associated with the severity of AMS. The expression differences of these eight genes among different groups are shown in Fig. 4b. Besides, Fig. 4c shows the correlation between the candidate genes.

### Functional annotation and pathway enrichment analysis of candidate genes

**Fig. 5:**
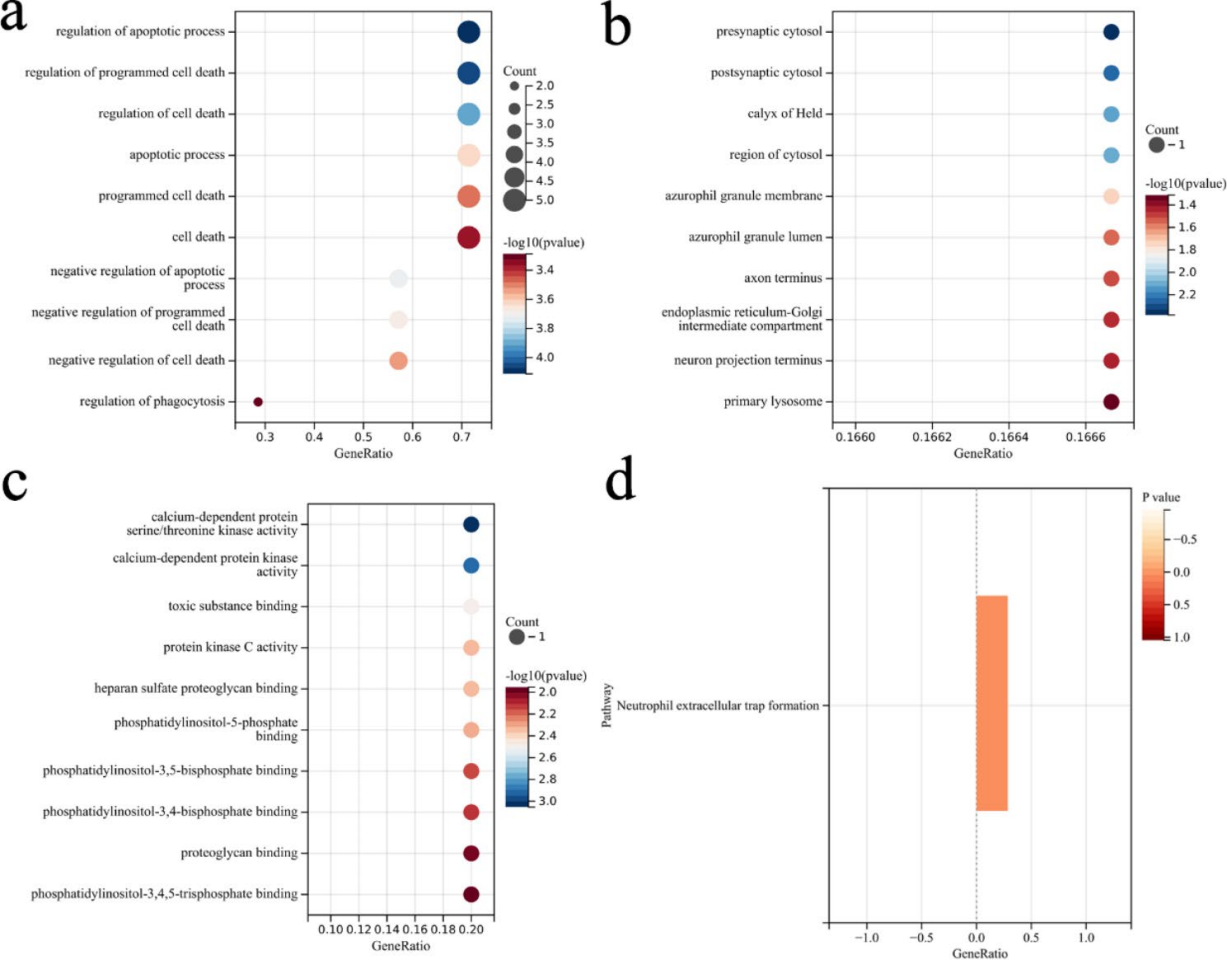
Functional Annotation and Pathway Enrichment Analysis of Candidate Genes. (a, b, c) The GO enrichment analysis of the candidate genes. Bubble chart showing the top terms in BP, CC, and MF groups. (d) The term for KEGG pathway analysis of candidate genes is shown with bar graphs. GO, gene ontology; KEGG, kyoto encyclopedia of genes and genomes; BP, biological process; CC, cell component; MF, molecular function

To further understand the biological functions in which these genes are involved, we performed enrichment analysis. The GO enrichment analysis included the following three portions: biological process (BP), cell component (CC), and molecular function (MF). The primary biological roles of candidate genes include regulation of apoptotic process (GO:0042981), regulation of programmed cell death (GO:0043067), regulation of cell death (GO:0010941), negative regulation of apoptotic process (GO:0043066), and calcium-dependent protein serine/threonine kinase activity (GO:0009931). As Fig. 5a, b, and c show. The candidate genes were primarily enriched in pathways associated with hsa04613: Neutrophil extracellular trap formation (Fig. 5d). Detailed information on these results is available in Table S4 ~ 5.

### ROC curves of candidate genes and correlation analysis

**Fig. 6:**
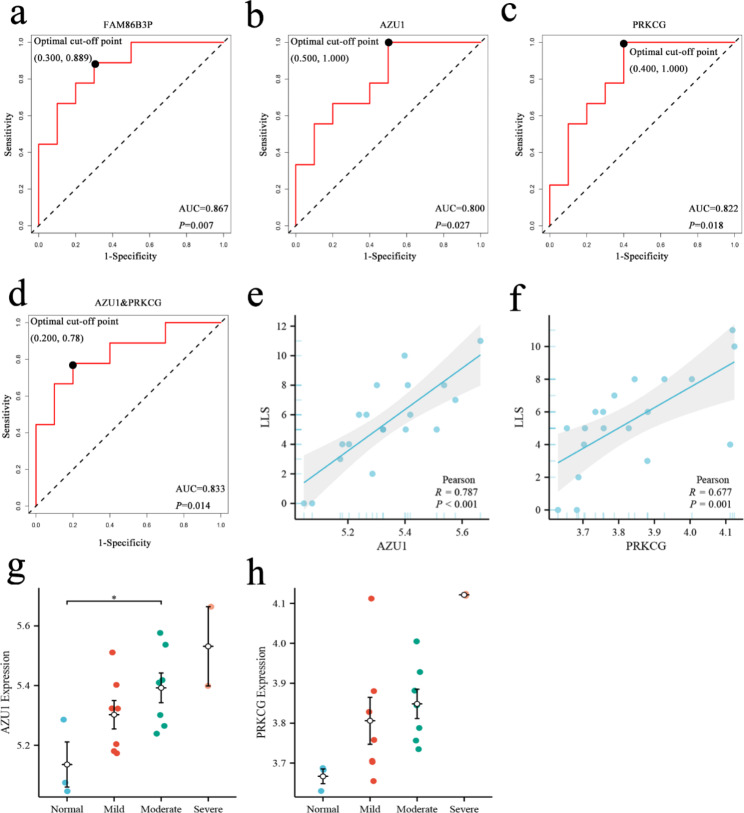
ROC Curves of Candidate Genes (a) ROC curve of FAM86B3P to predict MS-AMS. (b) ROC curve of AZU1 to predict MS-AMS. (c) ROC curve of PRKCG to predict MS-AMS. (d) ROC curve of AZU1 together with PRKCG. € Correlation analysis of AZU1 with LLS. (f) Correlation analysis of PRKCG with LLS. (g) AZU1 expression in the blood of patients with varying severity of AMS. (h) PRKCG expression in the blood of patients with varying severity of AMS. ROC curve, receiver operating characteristic curve

To explore the performance of these eight genes in predicting MS-AMS and to identify more critical candidate genes, we made ROC curves for each of the eight genes. ROC curve analysis showed that FAM86B3P (AUC = 0.867, *P* = 0.007), AZU1 (AUC = 0.800, *P* = 0.027) and PRKCG (AUC = 0.822, *P* = 0.018) were good predictors of moderate to severe AMS. By analyzing the optimal cut-off point (at the maximum of the Youden index), AZU1 expression at 5.221 was able to distinguish NM-AMS and MS-AMS sensitively with a sensitivity of 100% and specificity of 50%. PRKCG expression of 3.720 was able to distinguish NM-AMS and MS-AMS with sensitivity of 100% and specificity of 60%. Although FAM86B3P was a good predictor of moderate to severe AMS, FAM86B3P was a pseudogene and therefore excluded, as we will mention in the Discussion section. We then tried to explore the ability of AZU1 and PRKCG to predict MS-AMS together, and the results showed that the joint prediction had better predictive power than the separate prediction (AUC = 0.833, *P* = 0.014). The sensitivity at the optimal cut-off point was 78.8% and the specificity was 80%. The ROC curve is shown in Fig. 6a, b, c, and d. For more information on the ROC curves for other genes, see Table S6.

AZU1 (r = 0.787, *P* < 0.001) and PRKCG (r = 0.677, *P* = 0.001) were significantly correlated with LLS, as shown in Fig. 6e, f. Meanwhile, the expression of AZU1 and PRKCG in the blood of patients with different severity of AMS is shown in Fig. 6g, h. It should be noted that only two subjects entered the severe group according to the LLS grouping, and this group did not participate in the One-way ANOVA test. But it still can be clearly seen that the expression of AZU1 or PRKCG increases with the increase of AMS severity. In conclusion, these analyses suggest that AZU1 and PRKCG are associated with AMS severity and are good predictors of moderate to severe AMS.

### Verification by another grouping method, another dataset, real-time quantitative PCR

**Fig. 7:**
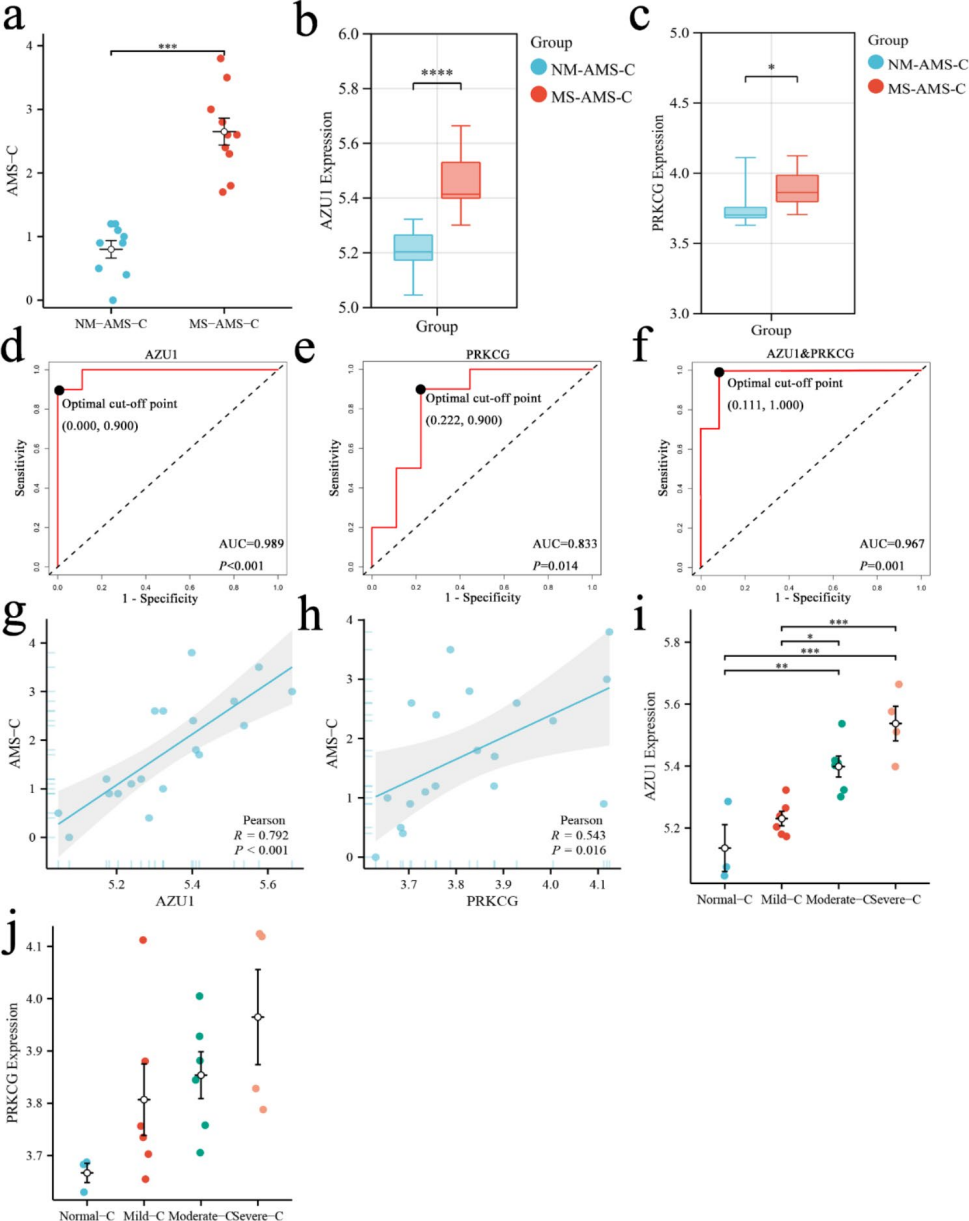
The analysis results of the LLS subgroup are validated by the AMS-C subgroup. (a) AMS-C differences between the NM-AMS-C and MS-AMS-C groups. (b) The difference in AZU1 expression between the two groups. (c) The difference in PRKCG expression between the two groups. (d) ROC curve analysis of AZU1 predicted MS-AMS-C. (e) ROC curve analysis of PRKCG predicted MS-AMS-C. (f) ROC curve analysis of AZU1&PRKCG predicted MS-AMS-C. (g) Correlation of AZU1 with AMS-C. (h) Correlation of PRKCG with AMS-C. (i) AZU1 expression in the blood of patients with varying degrees of AMS. (j) PRKCG expression in the blood of patients with varying degrees of AMS

We validated the results of this study using an alternative method of diagnosing AMS. The study subjects were divided into NM-AMS-C and MS-AMS-C groups according to AMS-C, as detailed in Sect. 2.3 and Table 2. There was a significant difference in the AMS-C scores between the two groups (Fig. 7a). All phenotypic and clinical data were not significantly different between MS-AMS-C group and NM-AMS-C group, as detailed in Table S7, 8. This is consistent with our previous analysis.

AZU1 and PRKCG expressions were significantly different between the NM-AMS-C and MS-AMS-C groups and had higher expression levels in the moderate to severe AMS group, as shown in Fig. 7b, c. ROC curve analysis showed that AZU1 (AUC = 0.989, *P* < 0.001) and PRKCG (AUC = 0.833, *P* = 0.014) were still good predictors of moderate to severe AMS. AZU1 of 5.323 predicted moderate to severe AMS with 90% sensitivity and 100% specificity (Fig. 7d). The PRKCG was 3.757, with a sensitivity of 90% and specificity of 77.8% for predicting moderate to severe AMS (Fig. 7e). Combined AZU1 and PRKCG also predicted moderate to severe AMS well (Fig. 7f). This is consistent with the results of our study.

Correlation analysis showed that AZU1 and PRKCG expression were significantly correlated with AMS-C, as shown in Fig. 7g, h. Besides, the expression levels of both AZU1 and PRKCG increased with the increase of AMS severity. This result can be seen in Fig. 7i, j. This result is also consistent with our analysis that AZU1 and PRKCG are associated with AMS severity.

**Fig. 8:**
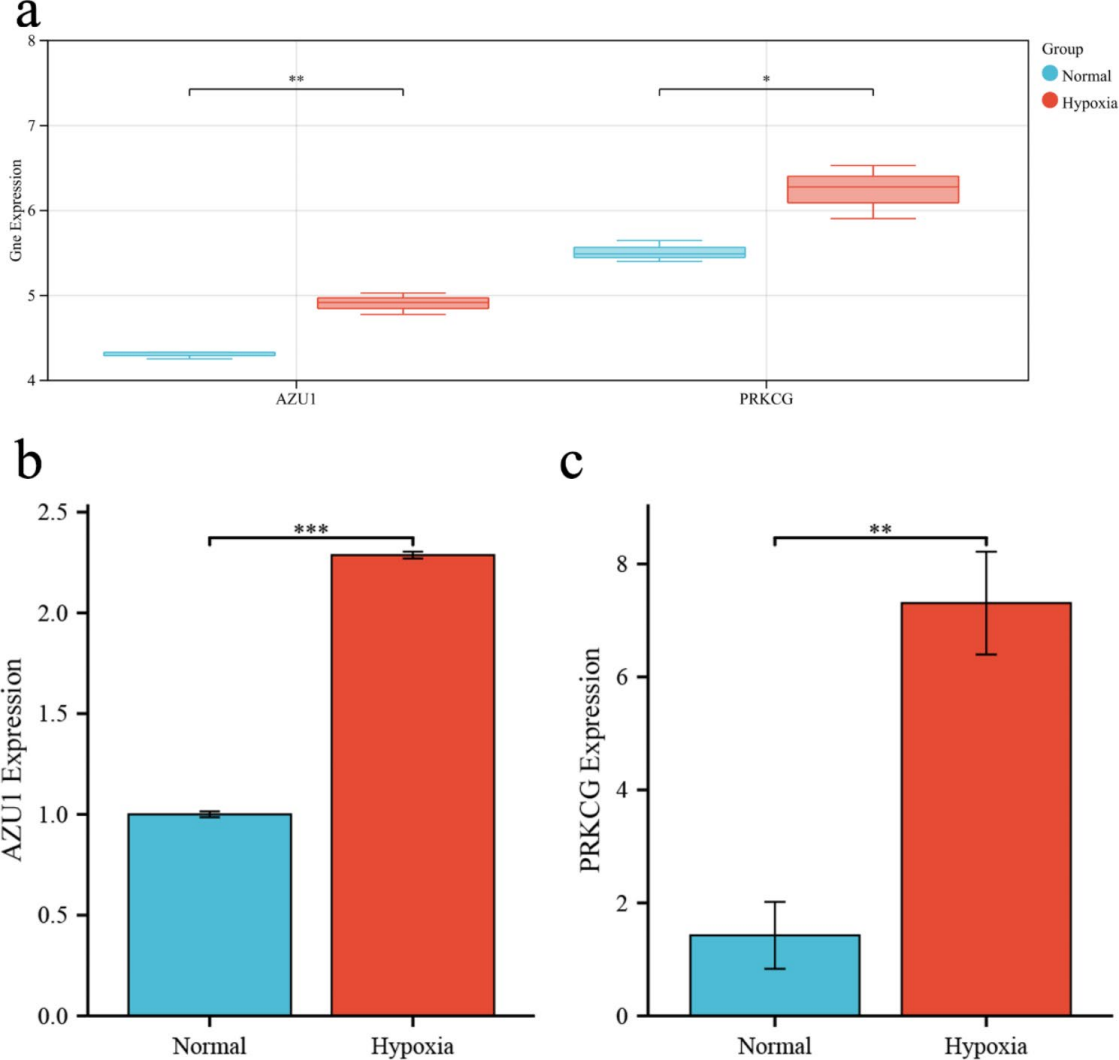
Transcript levels of AZU1 and PRKCG in GSE145935 and hypobaric hypoxia environment. (A) Transcript levels of candidate genes in GSE145935. (B) Transcript levels of AZU1 under hypobaric hypoxia treatment. (C) Transcript levels of PRKCG under hypobaric hypoxia treatment. *, *P* < 0.050; **, *P* < 0.010; ***, *P* < 0.001

Finally, we verified the changes in the expression of AZU1 and PRKCG under hypobaric hypoxia environment. GSE145935, another dataset in the GEO database, was used for validation (Fig. 8a). We also performed real-time quantitative PCR to detect the expression of candidate genes under hypobaric hypoxic conditions. The results showed that the transcript levels of AZU1 and PRKCG were increased under hypobaric hypoxic conditions **(**Fig. 8b and c).

## Discussion

In this study, dataset GSE103927 was downloaded from the GEO database. According to the 2018 edition of the LLS, the research subjects were divided into the NM-AMS group and the MS-AMS group. The differences in phenotypes and genes between the two groups at H1 were analyzed. The differences in phenotypic and clinical data between the two groups were not significant, suggesting the importance of gene expression. The modules and key genes most associated with LLS were obtained by WGCNA analysis. The intersection of differentially expressed genes with key genes obtained by WGCNA was selected as a candidate gene for AMS. To explore their biological function, these candidate genes were enriched for GO and KEGG function. ROC curves were made to evaluate the predictive performance of the three candidate genes. AZU1 and PRKCG are good predictors of moderate to severe AMS. We validated the results of the analysis by regrouping the subjects using AMS-C. Hypobaric hypoxia environment promotes AZU1 and PRKCG expression. In conclusion, AZU1 and PRKCG were associated with the severity of AMS.

It is important to note that in this study, subjects used two separate grouping methods, the LLS and the AMS-C. The AMS-C is an item in the ESQ questionnaire that has been used several times for the diagnosis of AMS [13–15]. We first grouped and compared the subjects using the LLS criteria and later grouped them again using the AMS-C, which was used to validate the results obtained from the LLS group comparison analysis. This eliminated some bias and made the results of this study more convincing.

We tried to find variables that discriminate moderate to severe AMS from other subjects, and phenotype was the primary consideration because phenotype is more likely to be of interest. Therefore, we first compared phenotypes between the MS-AMS group and the NM-AMS group. However, there were no statistically significant differences in phenotypic data between patients with no or mild AMS and moderate to severe AMS, regardless of which grouping was used to group the subjects. This suggests to us that the differences between moderate to severe AMS and no or mild AMS in the early stages of AMS may be due to differences in gene expression.

After DEGs analysis and WGCNA analysis, eight genes were identified as key genes because they were associated with LLS and differentially expressed between the two groups. These eight genes are FAM86B3P, AZU1, PHLDA3, MYDGF, RIPPLY3, PRKCG, CAAP1 and ZNF45. FAM86B3P is a pseudogene and little research has been done on FAM86B3P. Pseudogenes are variant copies of protein-coding genes that cannot be translated into proteins and rarely do. Therefore, it was excluded in the follow-up study. To gain insight into the biological roles of the eight key genes, we did an enrichment analysis. GO enrichment analysis suggested that these genes were associated with regulation of apoptotic process, regulation of programmed cell death, regulation of cell death, and calcium-dependent protein serine/threonine kinase activity. KEGG enrichment analysis showed that AZU1 and PRKCG were enriched to the Neutrophil extracellular trap formation pathway.

ROC curve analysis showed that AZU1 and PRKCG were good predictors of moderate to severe AMS. When subjects were grouped with LLS. The sensitivity of AZU1 to predict MS-AMS was 100% and the specificity was 50% according to the best cutoff analysis. the sensitivity when PRKCG was used as a predictor was 100% and the specificity was 60%. the sensitivity when AZU1 and PRKCG were combined as predictors was 78% and the specificity was 80%. When grouping with AMS-C. The best cut-off point showed a sensitivity of 90% and specificity of 100% for AZU1 to predict MS-AMS. 90% sensitivity and 77.8% specificity when PRKCG was used as a predictor. 100% sensitivity and 88.9% specificity when AZU1 and PRKCG were used together as predictors. The expression of AZU1 and PRKCG was proportional to LLS and AMS-C and increased with increasing AMS severity. These results suggest that the expression of AZU1 and PRKCG may influence the severity of AMS.

Azurocidin 1 (AZU1), also known as heparin-binding protein (HBP) or cationic antimicrobial protein of 37 KDa (CAP37), is a neutrophil-derived granule protein [47]. HBP is associated with several diseases and may be a new biomarker in sepsis [47]. Previous studies have shown that AZU1 is associated with hypoxic lung disease. The expression levels of AZU1in patients with acute lung injury (ALI) are higher than in patients without ALI. This phenomenon is also present in patients with acute respiratory distress syndrome (ARDS) [47–49]. Besides, the elevation of AZU1 is associated with a decrease in arterial oxygen partial pressure (PaO_2_) [50]. Protein kinase C gamma (PRKCG, also known as PKC-gamma, PKCγ, and SCA14) is located on chromosome 19 and encodes the γ isoform of the PKC family [51, 52]. PKC is a family of protein serine/threonine kinases composed of multiple isoforms that play essential roles in cell mitosis and proliferation, apoptosis, and platelet activation [53]. It has been shown that PRKCG is associated with brain disorders caused by hypoxia. PRKCG is involved in the hypoxia-induced reduction of blood-brain barrier permeability [54]. In the oxygen-glucose deprivation model, PKC-γ expression was associated with hypoxia-induced mitochondrial depolarization, increased ROS, and elevated calcium ions [55]. Our results also show that the hypobaric hypoxic environment promotes the expression of AZU1 and PRKCG.

Previous studies have shown that T cells or monocytes release AZU1 chemokines, such as IL-8, when in a hypoxic or infected state [56, 57]. Upon stimulation with IL-8, neutrophils release AZU1 preexisting in secretory vesicles and sulfur cell granules. released AZU1 acts on glycosaminoglycans on the surface of endothelial cells, activating PKC and Rho kinase, while allowing the entry of Ca^2+^. Hypoxia-generated ROS promote PLCγ expression and activate PKC together with Ca^2+^. These alterations rearrange the endothelial cytoskeleton, leading to increased endothelial permeability and inducing the onset of edema while leaving the possibility for neutrophils to act on other tissues and cells [47, 58]. These biological processes are likely to occur in the hypobaric hypoxia environment of high altitude [59].

In the present study, AZU1 and PRKCG were enriched to the Neutrophil extracellular trap formation pathway. The Neutrophil extracellular trap formation pathway has been shown to be associated with lung injury in diseases such as influenza and sepsis [60–62]. There have been many studies shows that the HBP (AZU1) and PKC pathways play a role in the development of pulmonary edema, as gates lead to enhanced endothelial cell permeability [50, 63]. Therefore, it is reasonable to speculate that this is one of the causes of the development of high altitude pulmonary edema. However, this still requires more clinical samples and experimental validation, which is one of the future research directions.

In this study, we propose for the first time that AZU1 and PRKCG are associated with the severity of AMS, and that AZU1 and PRKCG may be key genes affecting the severity of AMS. Our study provides new insights into the mechanisms of AMS. However, this study also has some limitations such as lack validation from clinical samples and small sample size. Many publicly available datasets could not be applied to this study because of the lack of LLS or AMS-C to group the samples. The subjective nature of LLS may lead to some bias, and although we used AMS-C to validate the analysis results, no estimates were made for these biases. No further exploration of how AZU1 and PRKCG function in AMS or hypobaric hypoxia environments via the Neutrophil extracellular trap formation pathway was performed. Therefore, exploration in larger cohorts and experiments is still needed in the future.

## Conclusion

AZU1 (HBP or CAP37) and PRKCG (PKC-gamma) are associated with the severity of acute mountain sickness and may be key genes influencing the severity of acute mountain sickness. AZU1 and PRKCG can be used as good diagnostic or predictive indicators of the severity of AMS. This study helps elucidate Acute Mountain Sickness’s pathogenesis and the mechanisms affecting its severity. Our study provides a new perspective on it. Inhibition or targeting of these genes may improve the health effects of the high-altitude environment.

## Electronic supplementary material

Below is the link to the electronic supplementary material.

Supplementary Material 1

## Data Availability

The datasets analyzed during the current study are available in the Gene Expression Omnibus (GEO) repository, https://www.ncbi.nlm.nih.gov/geo/query/acc.cgi?acc=GSE103927.

## Abbreviations

AMS: acute mountain sickness
ARDS: acute respiratory distress syndrome
AUC: area under curve
BP: biological process
CC: cell component
cDNA: DNA complementary to RNA
DEGs: differentially expressed gene(s)
EPO: erythropoietin
GEO: gene expression omnibus database
GP: gene ontology
GS: gene significance
H1: he first day on high altitude
HAPE: high altitude pulmonary edema
HB: hemoglobin
HREs: hypoxia response element(s)
kDa: kilodalton(s)
KEGG: kyoto encyclopedia of genes and genomes
LLS: lake louise score
MAD: median absolute deviation
MF: molecular function
MM: modules membership
MS-AMS: moderate to severe AMS
NM-AM: mild or no AMS
ROC: receiver operating characteristic
qRT-PCR: real time quantitative PCR
SL: sea level
TOM: topological overlap matrix
V/Q: ventilation perfusion ratio
WGCNA: weighted gene co-expression network analysis; *, *P* < 0.050; **, *P* < 0.010; ***, *P* < 0.001.

## References

[1] Ding XH, Wang Y, Cui B, Qin J, Zhang JH, Rao RS, Yu SY, Zhao XH, Huang L. Acute Mountain Sickness Is Associated With a High Ratio of Endogenous Testosterone to Estradiol After High-Altitude Exposure at 3,700 m in Young Chinese Men. *Front Physiol* 2018, 9:1949. 10.3389/fphys.2018.01949 PMC6355701 30740062

[2] Liu C, Liu B, Liu L, Zhang EL, Sun BD, Xu G, Chen J, Gao YQ (2018). Arachidonic acid metabolism pathway is not only Dominant in Metabolic Modulation but Associated with phenotypic variation after Acute Hypoxia exposure. Front Physiol.

[3] Hackett PH, Roach RC (2001). High-altitude illness. N Engl J Med.

[4] Gonggalanzi L, Nafstad P, Stigum H, Wu T, Haldorsen OD, Ommundsen K, Bjertness E (2016). Acute mountain sickness among tourists visiting the high-altitude city of Lhasa at 3658 m above sea level: a cross-sectional study. Arch Public Health.

[5] Johnson NJ, Luks AM (2016). High-Altitude Medicine. Med Clin North Am.

[6] Sun K, Zhang Y, D’Alessandro A, Nemkov T, Song A, Wu H, Liu H, Adebiyi M, Huang A, Wen YE (2016). Sphingosine-1-phosphate promotes erythrocyte glycolysis and oxygen release for adaptation to high-altitude hypoxia. Nat Commun.

[7] Liu B, Huang H, Wu G, Xu G, Sun BD, Zhang EL, Chen J, Gao YQ (2017). A signature of circulating microRNAs predicts the susceptibility of Acute Mountain sickness. Front Physiol.

[8] Hancco I, Bailly S, Baillieul S, Doutreleau S, Germain M, Pepin JL, Verges S (2020). Excessive erythrocytosis and Chronic Mountain sickness in dwellers of the Highest City in the World. Front Physiol.

[9] San Martin R, Brito J, Siques P, Leon-Velarde F (2017). Obesity as a conditioning factor for High-Altitude Diseases. Obes Facts.

[10] Hughes BH, Brinton JT, Ingram DG, Halbower AC. The Impact of Altitude on Sleep-Disordered Breathing in Children Dwelling at High Altitude: A Crossover Study.Sleep2017, 40(9). 10.1093/sleep/zsx120 PMC5806567 28934528

[11] Bourdillon N, Fan JL, Uva B, Muller H, Meyer P, Kayser B (2015). Effect of oral nitrate supplementation on pulmonary hemodynamics during exercise and time trial performance in normoxia and hypoxia: a randomized controlled trial. Front Physiol.

[12] Fan X, Ma L, Zhang Z, Li Y, Hao M, Zhao Z, Zhao Y, Liu F, Liu L, Luo X (2018). Associations of high-altitude polycythemia with polymorphisms in PIK3CD and COL4A3 in tibetan populations. Hum Genomics.

[13] Sweeting AJ, Billaut F, Varley MC, Rodriguez RF, Hopkins WG, Aughey RJ (2017). Variations in Hypoxia impairs muscle oxygenation and performance during simulated Team-Sport running. Front Physiol.

[14] Jiang X, Tian W, Tu AB, Pasupneti S, Shuffle E, Dahms P, Zhang P, Cai H, Dinh TT, Liu B (2019). Endothelial hypoxia-inducible Factor-2alpha is required for the maintenance of Airway Microvasculature. Circulation.

[15] Yue X, Lin X, Yang T, Yang X, Yi X, Jiang X, Li X, Li T, Guo J, Dai Y (2016). Rnd3/RhoE modulates hypoxia-inducible factor 1alpha/Vascular endothelial growth factor signaling by stabilizing hypoxia-inducible factor 1alpha and regulates responsive Cardiac Angiogenesis. Hypertension.

[16] Wang T, Shi F, Wang J, Liu Z, Su J (2017). Kallistatin suppresses Cell Proliferation and Invasion and promotes apoptosis in Cervical Cancer through blocking NF-kappaB signaling. Oncol Res.

[17] Huang J, Tang L, Zhao Y, Ding W (2019). TRIM11 promotes tumor angiogenesis via activation of STAT3/VEGFA signaling in lung adenocarcinoma. Am J Cancer Res.

[18] Krauszman A, Mak TW, Szaszi K, Kuebler WM (2017). Role of phosphatase and tensin homolog in hypoxic pulmonary vasoconstriction. Cardiovasc Res.

[19] Schodel J, Grampp S, Maher ER, Moch H, Ratcliffe PJ, Russo P, Mole DR (2016). Hypoxia, hypoxia-inducible transcription factors, and Renal Cancer. Eur Urol.

[20] Fidoamore A, Cristiano L, Antonosante A, d’Angelo M, Di Giacomo E, Astarita C, Giordano A, Ippoliti R, Benedetti E, Cimini A. Glioblastoma Stem Cells Microenvironment: The Paracrine Roles of the Niche in Drug and Radioresistance. *Stem Cells Int* 2016, 2016:6809105. 10.1155/2016/6809105 PMC4736577 26880981

[21] Roach RC, Hackett PH, Oelz O, Bartsch P, Luks AM, MacInnis MJ, Baillie JK, Lake Louise AMSSCC (2018). The 2018 Lake Louise Acute Mountain sickness score. High Alt Med Biol.

[22] Hufner K, Brugger H, Kuster E, Dunsser F, Stawinoga AE, Turner R, Tomazin I, Sperner-Unterweger B (2018). Isolated psychosis during exposure to very high and extreme altitude - characterisation of a new medical entity. Psychol Med.

[23] Moore J, MacInnis MJ, Dallimore J, Wilkes M (2020). The Lake Louise score: a critical Assessment of its specificity. High Alt Med Biol.

[24] Richalet JP, Julia C, Lhuissier FJ (2021). Evaluation of the Lake Louise score for Acute Mountain sickness and its 2018 version in a cohort of 484 Trekkers at High Altitude. High Alt Med Biol.

[25] Talks BJ, Campbell C, Larcombe SJ, Marlow L, Finnegan SL, Lewis CT, Lucas SJE, Harrison OK, Pattinson KTS (2022). Baseline psychological traits contribute to Lake Louise Acute Mountain sickness score at high Altitude. High Alt Med Biol.

[26] Song A, Zhang Y, Han L, Yegutkin GG, Liu H, Sun K, D’Alessandro A, Li J, Karmouty-Quintana H, Iriyama T (2017). Erythrocytes retain hypoxic adenosine response for faster acclimatization upon re-ascent. Nat Commun.

[27] Smirl JD, Lucas SJ, Lewis NC, duManoir GR, Smith KJ, Bakker A, Basnyat AS, Ainslie PN (2014). Cerebral pressure-flow relationship in lowlanders and natives at high altitude. J Cereb Blood Flow Metab.

[28] Willmann G, Fischer MD, Schommer K, Bartsch P, Gekeler F, Schatz A (2014). Missing correlation of retinal vessel diameter with high-altitude headache. Ann Clin Transl Neurol.

[29] Zhang X, Sun Y, Wang P, Yang C, Li S (2017). Exploration of the molecular mechanism of prostate cancer based on mRNA and miRNA expression profiles. Onco Targets Ther.

[30] Subudhi AW, Bourdillon N, Bucher J, Davis C, Elliott JE, Eutermoster M, Evero O, Fan JL, Jameson-Van Houten S, Julian CG (2014). AltitudeOmics: the integrative physiology of human acclimatization to hypobaric hypoxia and its retention upon reascent. PLoS ONE.

[31] Blankers M, Koeter MW, Schippers GM (2010). Missing data approaches in eHealth research: simulation study and a tutorial for nonmathematically inclined researchers. J Med Internet Res.

[32] Vaden KI, Gebregziabher M, Dyslexia Data C, Eckert MA (2020). Fully synthetic neuroimaging data for replication and exploration. NeuroImage.

[33] Donders AR, van der Heijden GJ, Stijnen T, Moons KG (2006). Review: a gentle introduction to imputation of missing values. J Clin Epidemiol.

[34] Young BA, Katz R, Boulware LE, Kestenbaum B, de Boer IH, Wang W, Fulop T, Bansal N, Robinson-Cohen C, Griswold M (2016). Risk factors for Rapid kidney function decline among African Americans: the Jackson Heart Study (JHS). Am J Kidney Dis.

[35] Howell NA, Tu JV, Moineddin R, Chu A, Booth GL (2019). Association between Neighborhood Walkability and predicted 10-Year Cardiovascular Disease Risk: the CANHEART (Cardiovascular Health in Ambulatory Care Research Team) Cohort. J Am Heart Assoc.

[36] Hunt JS, Theilmann RJ, Smith ZM, Scadeng M, Dubowitz DJ (2013). Cerebral diffusion and T(2): MRI predictors of acute mountain sickness during sustained high-altitude hypoxia. J Cereb Blood Flow Metab.

[37] Sibomana I, Foose DP, Raymer ML, Reo NV, Karl JP, Berryman CE, Young AJ, Pasiakos SM, Mauzy CA (2021). Urinary metabolites as predictors of Acute Mountain Sickness Severity. Front Physiol.

[38] Ritchie ME, Phipson B, Wu D, Hu Y, Law CW, Shi W, Smyth GK (2015). Limma powers differential expression analyses for RNA-sequencing and microarray studies. Nucleic Acids Res.

[39] Xie F, Wang Q, Sun R, Zhang B (2015). Deep sequencing reveals important roles of microRNAs in response to drought and salinity stress in cotton. J Exp Bot.

[40] Shen W, Song Z, Zhong X, Huang M, Shen D, Gao P, Qian X, Wang M, He X, Wang T et al. Sangerbox: A comprehensive, interaction-friendly clinical bioinformatics analysis platform. *iMeta*, n/a(n/a):e36. 10.1002/imt2.36 PMC10989974 38868713

[41] Langfelder P, Horvath S (2008). WGCNA: an R package for weighted correlation network analysis. BMC Bioinformatics.

[42] Tang J, Kong D, Cui Q, Wang K, Zhang D, Gong Y, Wu G (2018). Prognostic genes of breast Cancer identified by gene co-expression network analysis. Front Oncol.

[43] Yu G, Wang LG, Han Y, He QY (2012). clusterProfiler: an R package for comparing biological themes among gene clusters. OMICS.

[44] Kanehisa M, Goto S (2000). KEGG: kyoto encyclopedia of genes and genomes. Nucleic Acids Res.

[45] Kanehisa M (2019). Toward understanding the origin and evolution of cellular organisms. Protein Sci.

[46] Kanehisa M, Furumichi M, Sato Y, Kawashima M, Ishiguro-Watanabe M (2023). KEGG for taxonomy-based analysis of pathways and genomes. Nucleic Acids Res.

[47] Fisher J, Linder A (2017). Heparin-binding protein: a key player in the pathophysiology of organ dysfunction in sepsis. J Intern Med.

[48] Johansson J, Brattstrom O, Sjoberg F, Lindbom L, Herwald H, Weitzberg E, Oldner A (2013). Heparin-binding protein (HBP): an early marker of respiratory failure after trauma?. Acta Anaesthesiol Scand.

[49] Lin Q, Shen J, Shen L, Zhang Z, Fu F (2013). Increased plasma levels of heparin-binding protein in patients with acute respiratory distress syndrome. Crit Care.

[50] Bentzer P, Fisher J, Kong HJ, Morgelin M, Boyd JH, Walley KR, Russell JA, Linder A (2016). Heparin-binding protein is important for vascular leak in sepsis. Intensive Care Med Exp.

[51] Sailer A, Scholz SW, Gibbs JR, Tucci A, Johnson JO, Wood NW, Plagnol V, Hummerich H, Ding J, Hernandez D (2012). Exome sequencing in an SCA14 family demonstrates its utility in diagnosing heterogeneous diseases. Neurology.

[52] Zhang Y, Xu J, Zhu X (2018). A 63 signature genes prediction system is effective for glioblastoma prognosis. Int J Mol Med.

[53] Gorman D, Lin HY, Williams C (2006). Early evidence of a regulated response to hypoxaemia in sheep that preserves the brain cortex. Neurosci Lett.

[54] Fleegal MA, Hom S, Borg LK, Davis TP (2005). Activation of PKC modulates blood-brain barrier endothelial cell permeability changes induced by hypoxia and posthypoxic reoxygenation. Am J Physiol Heart Circ Physiol.

[55] Surendran D (2019). PKCgamma and PKCepsilon are differentially activated and modulate neurotoxic signaling pathways during oxygen glucose deprivation in rat cortical slices. Neurochem Res.

[56] Le QT, Fisher R, Oliner KS, Young RJ, Cao H, Kong C, Graves E, Hicks RJ, McArthur GA, Peters L (2012). Prognostic and predictive significance of plasma HGF and IL-8 in a phase III trial of chemoradiation with or without tirapazamine in locoregionally advanced head and neck cancer. Clin Cancer Res.

[57] Chertov O, Michiel DF, Xu L, Wang JM, Tani K, Murphy WJ, Longo DL, Taub DD, Oppenheim JJ (1996). Identification of defensin-1, defensin-2, and CAP37/azurocidin as T-cell chemoattractant proteins released from interleukin-8-stimulated neutrophils. J Biol Chem.

[58] Kuhn M (2012). Endothelial actions of atrial and B-type natriuretic peptides. Br J Pharmacol.

[59] Song S, Yao N, Yang M, Liu X, Dong K, Zhao Q, Pu Y, He X, Guan W, Yang N (2016). Exome sequencing reveals genetic differentiation due to high-altitude adaptation in the tibetan cashmere goat (Capra hircus). BMC Genomics.

[60] Narasaraju T, Yang E, Samy RP, Ng HH, Poh WP, Liew AA, Phoon MC, van Rooijen N, Chow VT (2011). Excessive neutrophils and neutrophil extracellular traps contribute to acute lung injury of influenza pneumonitis. Am J Pathol.

[61] Hawez A, Taha D, Algaber A, Madhi R, Rahman M, Thorlacius H (2022). MiR-155 regulates neutrophil extracellular trap formation and lung injury in abdominal sepsis. J Leukoc Biol.

[62] Madhi R, Rahman M, Taha D, Morgelin M, Thorlacius H (2019). Targeting peptidylarginine deiminase reduces neutrophil extracellular trap formation and tissue injury in severe acute pancreatitis. J Cell Physiol.

[63] Gautam N, Olofsson AM, Herwald H, Iversen LF, Lundgren-Akerlund E, Hedqvist P, Arfors KE, Flodgaard H, Lindbom L (2001). Heparin-binding protein (HBP/CAP37): a missing link in neutrophil-evoked alteration of vascular permeability. Nat Med.

