# Supplementary material for: AZU1 (HBP/CAP37) and PRKCG (PKC-gamma) may be candidate genes affecting the severity of acute mountain sickness
Source: BMC Med Genomics. 2023 Feb 20;16:28. doi: 10.1186/s12920-023-01457-3 (PMC9940399; doi:10.1186/s12920-023-01457-3)
Supplement: Supplementary file 1 — Supplementary Material 1 [file 12920_2023_1457_MOESM1_ESM.docx]

# Additional files

Additional file 1: **Figure S1.** The result for selecting power value.

Additional file 2: **Figure S2**. Module feature vector clustering diagram.

Additional file 3: **Table S1**. R code for the multiple interpolation method for estimating missing values.

Additional file 4: **Table S2**. The result of T-test of General Characteristics between the two groups at H1.

Additional file 5: **Table S3**. The result of T-test of Clinical Data between the two groups at H1.

Additional file 6: **Table S4**. The result of Function Enrichment of key genes.

Additional file 6: **Table S5**. The result of KEGG Pathway Enrichment of key genes.

Additional file 6: **Table S6**. The details of ROC Curves of candidate genes.

Additional file 6: **Table S7**. The result of T-test of General Characteristics between NM-AMS-C group and MS-AMS-C group at H1.

Additional file 6: **Table S8.** The result of T-test of Clinical Data between NM-AMS-C group and MS-AMS-C group at H1.

**Figure S1** The result for selecting power value.


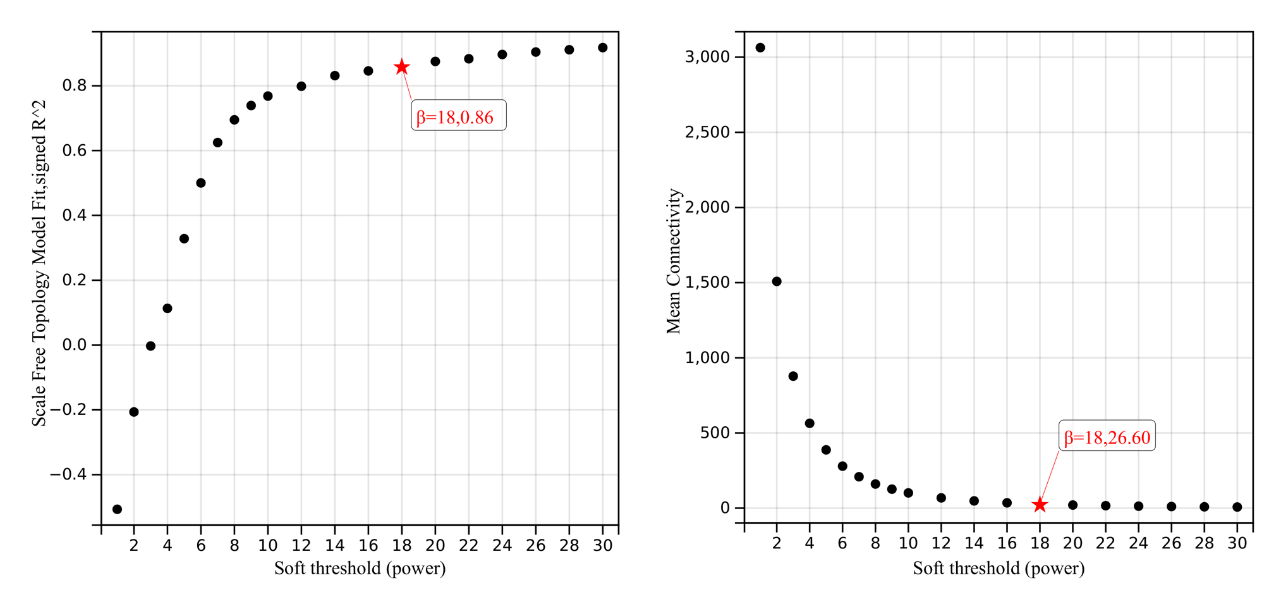


**Figure S2** Module feature vector clustering diagram.

**
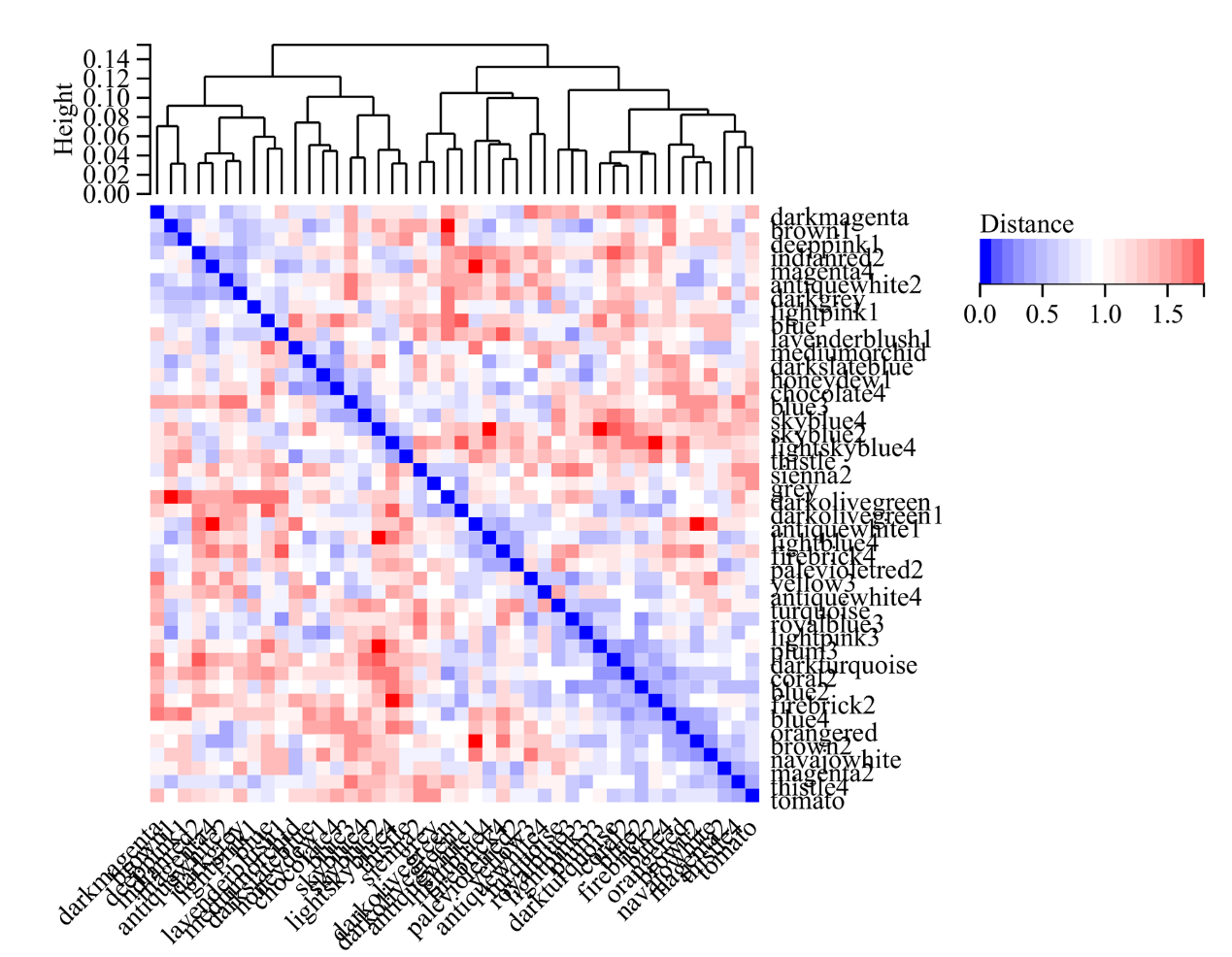
**
